# Transient Asystole after Sugammadex Administration for Immediate Reversal of Deep Blockade while on Dexmedetomidine Infusion in a Super Obese Patient

**DOI:** 10.1155/2019/2709568

**Published:** 2019-05-21

**Authors:** Michal Gajewski, Sorochi Esochaghi

**Affiliations:** Department of Anesthesiology, Rutgers New Jersey Medical School, USA

## Abstract

Sugammadex is increasingly used to reverse aminosteroid neuromuscular blocking agents. Dosing is calculated based on actual body weight, even for those who are obese. We report a case where a super obese patient (BMI 58.5 kg/m^2^) developed asystole, following coadministration with dexmedetomidine, for rapid reversal after deep blockade. Although 16mg/kg of actual body weight is recommended for prompt reversal of deep blockade, dosing adjustments may be prudent in the obese population, especially when used in conjunction with other negative chronotropic agents.

## 1. Introduction

Neuromuscular blockade during surgery improves surgical conditions and facilitates intubation and mechanical ventilation. In order to prevent residual neuromuscular blockade reversal is required when utilizing nondepolarizing blockers. If using traditional anticholinesterases, such as neostigmine, reversal is recommended only after the patient has regained at least two of four twitches in the train-of-four (TOF) response, which can take 30-40 minutes [1]. The introduction of sugammadex as a neuromuscular blockade reversal agent, presented an alternate option for practitioners, especially when requiring rapid reversal. With this drug, reversal is likely within 2-3 minutes and is possible immediately after full blockade, albeit at higher doses [1]. Its use has quickly become widespread but many anesthetists might not be aware of the adjustments which may be warranted when dosing the drug in the obese population.

Obese patients are at an increased risk of intraoperative anesthesia-related complications such as critical respiratory events, oversedation, hypoxia, and hypercapnia [2]. Dexmedetomidine has been increasingly advocated for use in the obese population specifically due to its analgesics and narcotic sparing effects [3]. This alpha-2 adrenergic agonist is however associated with an increased blood pressure and reflex bradycardia during the initial loading, while continuing administration results in inhibition of the central sympathetic system leading to hypotension and bradycardia [4]. As with sugammadex, dosing of dexmedetomidine is recommended according to actual body weight, possibly leading to exaggerated negative chronotropic effect.

Written informed consent for writing this case report was obtained from the patient.

## 2. Case Description

A 1.5m, 50-year-old female weighing 136.1kg (BMI 58.5 kg/m^2^) presented for a videolaryngoscopy with bronchoscopy and T-tube exchange. She had a history of type 2 diabetes mellitus, obstructive sleep apnea, hypothyroidism, and lymphoma. Following chemotherapy and radiation therapy, the patient developed tracheomalacia and tracheal stenosis, rendering her T-tube dependent. Preoperatively, the patient's vitals were within normal limits and stable. She denied any known drug allergies.

The intraoperative electrocardiogram (ECG) was sinus rhythm. Given the uncertainty by the surgical team regarding the potential difficulty of exchange, the decision was made to start the case under sedation with dexmedetomidine. A loading dose of dexmedetomidine was given (1 mcg/kg) over a period of 10 minutes, without any hemodynamic effects, and was then continued at 0.4 mcg/kg/h. Once the patient was rendered unresponsive to verbal stimuli, the T-tube exchange was attempted. As the level of anesthesia was still insufficient for the exchange, deepening of the anesthetic was requested. The anesthesia circuit was connected to the T-tube and sevoflurane was administered at 0.5 minimum alveolar concentration (MAC). As a subsequent attempt at exchange was still unsuccessful due to inadequate anesthesia, the surgeon requested muscular paralysis. The dexmedetomidine infusion was therefore stopped, the inhalational anesthetic was increased to 1 MAC, and 50 mg of rocuronium was administered to the patient. Within 5 minutes the T-tube was successfully exchanged and confirmed with direct laryngoscopy.

Upon termination of the surgical procedure, a peripheral nerve stimulator was used to evaluate the patient's depth of muscle paralysis. TOF stimulation of the ulnar nerve revealed 0/0 twitches with no recovery noted after tetany. In order to achieve complete reversal promptly, a sugammadex dose was calculated at 16 mg/kg, totaling 2177.6 mg. Due to our limited experience with this large dose, 1200 mg (55% of the calculated dose) was administered instead. Within about 30 seconds of the drug administration, it was noted that that the patient's heart rate precipitously decreased to 35 bpm and was immediately followed by asystole. At this point the surgeons were alerted of the sudden change in hemodynamic status; however prior to commencing chest compressions, the patient's heart rate spontaneously rebounded to the 90s (about 15 seconds total). Shortly afterwards, the patient began breathing spontaneously and TOF stimulation showed 4/4 twitches. The patient emerged from anesthesia and was transferred to the post anesthesia care unit (PACU) where a cardiology consult was obtained.

Postoperatively, the patient's 12 lead ECG showed normal sinus rhythm without any abnormalities. The patient was monitored overnight on telemetry with all blood work, including troponins, returning within normal limits. An echocardiogram showed no regional wall motion abnormalities and a preserved ejection fraction. The patient was successfully discharged home the following day.

## 3. Discussion

Sugammadex is a modified gamma cyclodextrin that irreversibly forms a complex with rocuronium and vecuronium preventing these aminosteroid neuromuscular blocking agents from acting at the nicotinic receptor [1]. Though some risks and adverse effects have been well elucidated, the use of sugammadex, including dosing regimens, has not been fully researched in the obese population. As the prescribing information from the manufacturer details, dosing is based on actual body weight and adverse effects may include marked bradycardia that could deteriorate to cardiac arrest [5]. A search of the medical literature reveals numerous case reports that have now been published reporting this [6, 7]. Our case further contributes to this finding with the added concern for dosing in the obese population. Although the true incidence of cardiac arrest from the use of sugammadex is unknown, worth noting is the significant increase in case reports to the FDA Adverse Event Reporting System (FAERS) compared to neostigmine. Since 1985, neostigmine has been reported for causing 121 serious cardiac adverse events, leading to 19 deaths, while in the span of 9 years, sugammadex has already been reported 138 times, 9 of which resulted in death [8].

The mechanism responsible for the bradycardia and asystole is unknown and case reports have noted such occurrence even at the prescribed low dose of 2mg/kg [6, 7]. In our patient, however, a much larger dose of sugammadex, 1200 mg, (8.8 mg/kg), was used, yet still below the recommended 16mg/kg of actual body weight indicated for prompt reversal. Although there is evidence suggesting that dosing according to ideal body weight for moderate neuromuscular blockade is effective, dosing of sugammadex according to actual body weight is still recommended for immediate reversal after deep blockade (16mg/kg) [9]. Adjusted body weight has also been studied when reversing deep blockade in the morbidly obese; however the dose used was only 4 mg/kg and due to the study's prospective observational design, further research is needed for conclusive results [10]. Similarly, Gaszynski et al. found successful reversal of neuromuscular blockade in the morbidly obese population based on corrected body weight; however this was after the reappearance of T2 in the TOF stimulation [11]. As such, a dose of 16 mg/kg is still recommended if there is a need to reverse the patient quickly after a single dose of 1.2 mg/kg of rocuronium irrespective of the patient's actual body weight. The manufacturer does not note an upper total limit for this regimen; hence no correlation can be made as to whether larger doses in obese patients cause an increased risk of adverse events.

Although the asystole in our patient is temporally related to the administration of sugammadex, a direct correlation cannot be made as dexmedetomidine was being concomitantly given. Despite the fact that no hemodynamic changes were noted during the initial bolus as well as the brief continued infusion, the effect can be noted for up to 5 to 10 minutes after the loading dose is given [4]. Additionally, as the drug exhibits a rapid distribution phase with a half-life of 6 minutes and a termination half-life of 2 hours, our patient's plasma concentration was most likely within the therapeutic range, possibly enhancing the negative chronotropic effects, which have been previously noted [12]. Regardless of these known adverse hemodynamic effects, dexmedetomidine is overwhelmingly advocated for its cardioprotective, neuroprotective, and opioid sparing qualities, especially in the obese population. Although no studies have specifically looked at hemodynamic effects of coadministering dexmedetomidine and sugammadex, case reports do suggest that it can be done safely [13, 14]. Only one report presents a case of severe bradycardia with the concomitant use of dexmedetomidine and sugammadex, however in a pediatric heart transplant patient where the physiology of the denervated heart may have played a role [15]. Hence, we cannot rule out the possibility that dexmedetomidine may have contributed to our patients episode.

As the use of sugammadex becomes more prevalent, it is important that practitioners be aware of the risks of cardiac events that can occur with this reversal agent, especially when using actual body weight when dosing in the obese population. Considering the evidence, as well as our own experience, when utilizing sugammadex for immediate reversal (16mg/kg), a dose based on measuring the twitch response, before and after tetany, as well as on ideal body weight may be more prudent. Similarly, coadministration with dexmedetomidine should be done with caution as both agents are known for their ability to precipitate bradycardia. As the incidence of bradycardia is seen with higher total doses, the provider should consider premedication with anticholinergics, especially when used concomitantly with negative chronotropic agents, and should be prepared to initiate advanced cardiac life support (ACLS) in the event of cardiac arrest.
